# The crucial role of HFM1 in regulating FUS ubiquitination and localization for oocyte meiosis prophase I progression in mice

**DOI:** 10.1186/s40659-024-00518-w

**Published:** 2024-05-31

**Authors:** Chenyi Zhong, Huiyuan Wang, Xiong Yuan, Yuheng He, Jing Cong, Rui Yang, Wenjie Ma, Li Gao, Chao Gao, Yugui Cui, Jie Wu, Rongrong Tan, Danhua Pu

**Affiliations:** 1 State Key Laboratory of Reproductive Medicine and Offspring Health, Department of Obstetrics and Gynecology, The First Affiliated Hospital of Nanjing Medical University/Jiangsu Province Hospital/Jiangsu Women and Children Health Hospital, Nanjing, 210036 China; 2 State Key Laboratory of Reproductive Medicine and Offspring Health, Center of Reproduction and Genetics, Affiliated Suzhou Hospital of Nanjing Medical University, Suzhou Municipal Hospital, Gusu School, Nanjing Medical University, Suzhou, 215002 China

**Keywords:** HFM1, Meiosis prophase I, Premature ovarian failure/insufficiency, Oocyte, FUS

## Abstract

**Background:**

Helicase for meiosis 1 (HFM1), a putative DNA helicase expressed in germ-line cells, has been reported to be closely associated with premature ovarian insufficiency (POI). However, the underlying molecular mechanism has not been clearly elucidated. The aim of this study was to investigate the function of HFM1 in the first meiotic prophase of mouse oocytes.

**Results:**

The results suggested that the deficiency of HFM1 resulting in increased apoptosis and depletion of oocytes in mice, while the oocytes were arrested in the pachytene stage of the first meiotic prophase. In addition, impaired DNA double-strand break repair and disrupted synapsis were observed in the absence of HFM1. Further investigation revealed that knockout of HFM1 promoted ubiquitination and degradation of FUS protein mediated by FBXW11. Additionally, the depletion of HFM1 altered the intranuclear localization of FUS and regulated meiotic- and oocyte development-related genes in oocytes by modulating the expression of BRCA1.

**Conclusions:**

These findings elaborated that the critical role of HFM1 in orchestrating the regulation of DNA double-strand break repair and synapsis to ensure meiosis procession and primordial follicle formation. This study provided insights into the pathogenesis of POI and highlighted the importance of HFM1 in maintaining proper meiotic function in mouse oocytes.

**Supplementary Information:**

The online version contains supplementary material available at 10.1186/s40659-024-00518-w.

## Background

Premature ovarian insufficiency (POI), a clinical syndrome, is characterized by menstrual disorders and reduced ovarian function in women before the age of 40 years [1, 2], which affects approximately 1% of women worldwide [3]. POI not only leads to female infertility but also poses long-term health risks for women, including bone health, cardiovascular system health, and cognitive function [4].

The etiology of POI is complex and heterogeneous, including genetic, autoimmune, and iatrogenic factors [5–8]. Patients with POI have a tendency to cluster in families, accounting for 5% to 10%, indicating genetic factor takes an important place in etiology [9–11]. Recently, a large number of POI candidate genes have been identified with the broad deployment of high-throughput microarray and sequencing technologies. Helicase for meiosis 1 (HFM1) is acknowledged as an ATP-dependent DNA helicase and has been identified as a candidate gene for POI in our previous studies [12, 13].

HFM1 is a putative DNA helicase of yeast and expressed in germ-line cells specifically [14]. A knockout (*Hfm1*-KO) mouse model used in a study found that *Hfm1*^*−/−*^ mice showed testicular hypoplasia and azoospermia in male mice, and had smaller ovaries and reduced numbers of follicle and corpus in female mice [15]. Further studies revealed that the deletion of *Hfm1* influenced cross-over formation and chromosome synapsis (tetrad formation), leading to impaired chromosome recombination during the first prophase of meiosis in spermatogenesis [15]. Because of the difference between the development patterns of spermatocytes and oocytes, the role of HFM1 in oogenesis and pathology of POI is not well understood.

Compound heterozygous mutations were identified in the *HFM1* gene by whole-exome sequencing in two POI sisters in our previous study [12]. Further exploration validated the association between *HFM1* mutation and POI by sequencing blood samples from Chinese patients with sporadic POI [13]. In addition, our previous study established a *Gdf9-Cre*-mediated oocyte conditional knockout mouse model of *Hfm1* gene (*Hfm1*-cKO) by CRISPR/Cas9 and showed that *Hfm1* was involved in Golgi-associated spindle assembly and division during oocyte meiosis I and II in mice, which indicating the profound impact of HFM1 in oocyte maturation [16]. However, the role of Hfm1 in the first meiotic prophase of oocytes was still to be studied. Distinct from the continuity of spermatogenesis in men, the oocyte enters the first meiotic prophase from 13.5 days post coitum (dpc) and undergoes four stages of leptotene, zygotene, pachytene, and diplotene, during which biological events such as chromosome synapsis and recombination occur. Subsequently, the oocytes are gradually arrested in the dictyotene stage (the terminal stage of the diplotene stage) perinatally. The arrested oocytes resume their first meiosis and continue to mature until the presence of pubertal gonadotropins [17, 18]. The meiotic progression is acknowledged to be related to the size of the primordial follicular pool, which is implicated in POI. Therefore, we would further explore the role of HFM1 in the first meiotic prophase of mouse oocytes in this study to explore the important implications of HFM1 in etiology of POI.

## Materials and methods

### Animals

The wild-type C57B6/J mice used in this study were purchased from Vital River Laboratory Animal Technology Co., Ltd (Beijing, China), and the *Hfm1*-cKO mice (*Hfm1*^*fl/fl*^*, Gdf9-Cre*) were generated by GemPharmatech Co., Ltd (Nanjing, China). All the mice were reared in a room with constant temperature and humidity, controlled the temperature at 21 ± 1 °C and the humidity at 60 ± 10%. All animals were provided with food and water ad libitum, with a 12/12 h light/dark alternating cycle. Furthermore, female mice aged 6 to 8 weeks were mated with male mice at the ratio of 1:1 overnight, the day of the presence of a vaginal plug was recorded as 0.5 days post coitum (dpc), and the day of the birth of pups was labeled as 1 dpp (day post-partum). The study protocol was approved and consented by the Animal Ethics Committee (AEC) of Nanjing Medical University (NMU) under the approval number IACUC-1911005.

### Ovary isolation and culture

The ovaries of 14.5 dpc were separated under a stereomicroscope (Olympus, Japan) under sterile conditions and cultured in individual wells of a six-well plate (NEST, China) with basic DMEM/F12 (Gibco, USA) at 37℃ with a 5% CO_2_ humidified atmosphere. The ovaries were treated with AD-*Hfm1i* (GeneChem, China) or AD-*Fus* (GenePharma, China) to alter the expression of related genes. The primers used for knockdown adenovirus HFM1 interfere in this assay are listed in Supplementary Table 3. The ovaries were injected with an adenovirus using capillary tubes under a stereomicroscope and then cultured in vitro. After 4 days of culture, the ovaries were collected for further experiments. The protease inhibitor MG132 was used to effectively block the proteolytic activity of the proteasome complex, and the protein synthesis inhibitor cycloheximide (CHX) was used to inhibit protein and RNA synthesis in the ovary culture system.

### Immunofluorescence

The ovaries were fixed in 4% paraformaldehyde (PFA) at 4 ℃ overnight, embedded in paraffin, and sliced into 5-μm-sections. After deparaffinization, rehydration and antigen retrieval were performed in 10 mM sodium citrate (pH 6.0; Beyotime, China) at a high temperature for 20 min. The sections were blocked with 5% goat serum at room temperature for 1 h and incubated with primary antibodies (diluted at 1:100 to 1:500) overnight at 4 °C. The primary antibodies used in the study are listed in Supplementary Table 2. After washing with phosphate-buffered saline (PBS), the sections were incubated with Alexa Fluor 488- or Alexa Fluor 594-conjugated donkey secondary antibody (1:100, Invitrogen, USA) at 37 °C for 1 h. Then, the sections were rinsed with PBS and stained with DAPI (Sigma–Aldrich, USA) for 15 min. Finally, the antifade mounting medium (Beyotime, China) was applied to each section. A confocal microscope (Nikon, Japan) was used for imaging immunofluorescent sections.

### Oocytes count

The 5-μm serial sections were stained with an antibody against germ cell-specific marker DEAD-Box Helicase 4 (DDX4) and nuclear marker DAPI, and the oocytes in every fifth section were counted. The totality was multiplied by 5 to estimate the total number of oocytes per ovary as previously described [19].

### TUNEL assay

The histological sections were prepared as described earlier. Apoptosis was detected for the 5-μm serial sections using a YF 488 TUNEL assay apoptosis detection kit (T6013, US Everbright, China) following the manufacturer’s protocols. TUNEL-positive cells were counted on each section.

### Chromatin spread and stain

The chromosomes were spread as previously described [20]. The sections were blocked with 5% goat serum at room temperature for 1 h and incubated with primary antibodies overnight at 4 °C. The primary antibodies and dilution rates are listed in Supplementary Table 2. The cells with relatively independent, clear signals and a low background were included for counting under a fluorescence microscope (Nikon, Japan), and this process was repeated in three mice.

### Immunoblotting

The ovaries were collected and extracted in RIPA or NP-40 cell lysis buffer (Beyotime, China) following the protocol. There were at least 15 embryonic ovaries in each sample. Then, the protein samples were separated on SDS-PAGE and transferred to polyvinylidene fluoride membranes (Millipore, USA). The membranes were incubated with appropriately diluted primary antibodies at 4 °C overnight. The primary antibodies used in the study are listed in Supplementary Table 2. The membranes were incubated with secondary antibodies diluted 1:5000 in TBST for 1 h at room temperature after thorough rinsing with TBST (TBS with 1% Tween 20). GAPDH, β-actin, or Vinculin was used as a loading control. Finally, the membranes were visualized using a Tanon 4800 Imaging System with ECL Ultra Reagent (NCM, China).

### Co-immunoprecipitation

The co-immunoprecipitation (Co-IP) assays were performed using Protein A/G magnetic beads (MCE, USA) following the manufacturer’s protocol. Then, 400 μL of the antibody was added to the pretreated magnetic beads and mixed at room temperature for 30 min, and the beads were collected by magnetic separation. The concentrations of antibodies referred to the recommendations of the manufacturer. Next, 17.5 dpc ovaries were digested and reacted with antibody-binding magnetic beads for 1 h at room temperature. The antigens were eluted, and the enriched proteins were analyzed by immunoblotting or mass spectrometry (MS).

### Transfection

Frozen HEK 293 T cells were recovered in a sterile water bath prewarmed at 37 °C. After the cells were grown and stabilized, the transfection of Flag-Hfm1 and Myc-Fus plasmids (dxgenes, China) was conducted following the instructions of the Lipofectamine 3000 kit (Thermo, USA), and the cells were collected after 48 or 72 h for further experiments.

### Real-time polymerase chain reaction (PCR)

Total RNA of ovaries was extracted using TRIzol reagent (Invitrogen, USA) and dissolved in diethyl pyrocarbonate (DEPC) treated water. Reverse transcription of total RNA was performed using a PrimeScript RT Master Mix Kit (TaKaRa, Japan). Quantitative polymerase chain reaction (PCR) was conducted on StepOnePlus Real-time PCR Systems (Applied Biosystems, USA) using the following parameters: pre-denaturation at 95℃ for 5 min, annealing at 95℃ for 3 s, and elongation at 60℃ for 30 s, with 40 cycles in total; the melting curve was detected in the end. The expression levels were normalized to β-actin expression. The primers used for analysis are listed in Supplementary Table 4.

### Statistical analysis

All the graphs and statistical analyses were generated using GraphPad Prism 8 (GraphPad Software, USA). All data were reported as mean ± standard deviation (SD). The student *t* test or one-way analysis of variance was used. Tukey’s multiple comparison test was used for further multiple comparisons. A minimum of three independent experiments were conducted for each experiment. A *P* value < 0.05 indicated a statistically significant difference.

## Results

### HFM1 was predominantly expressed in the prenatal embryonic mouse ovary

**Fig. 1:**
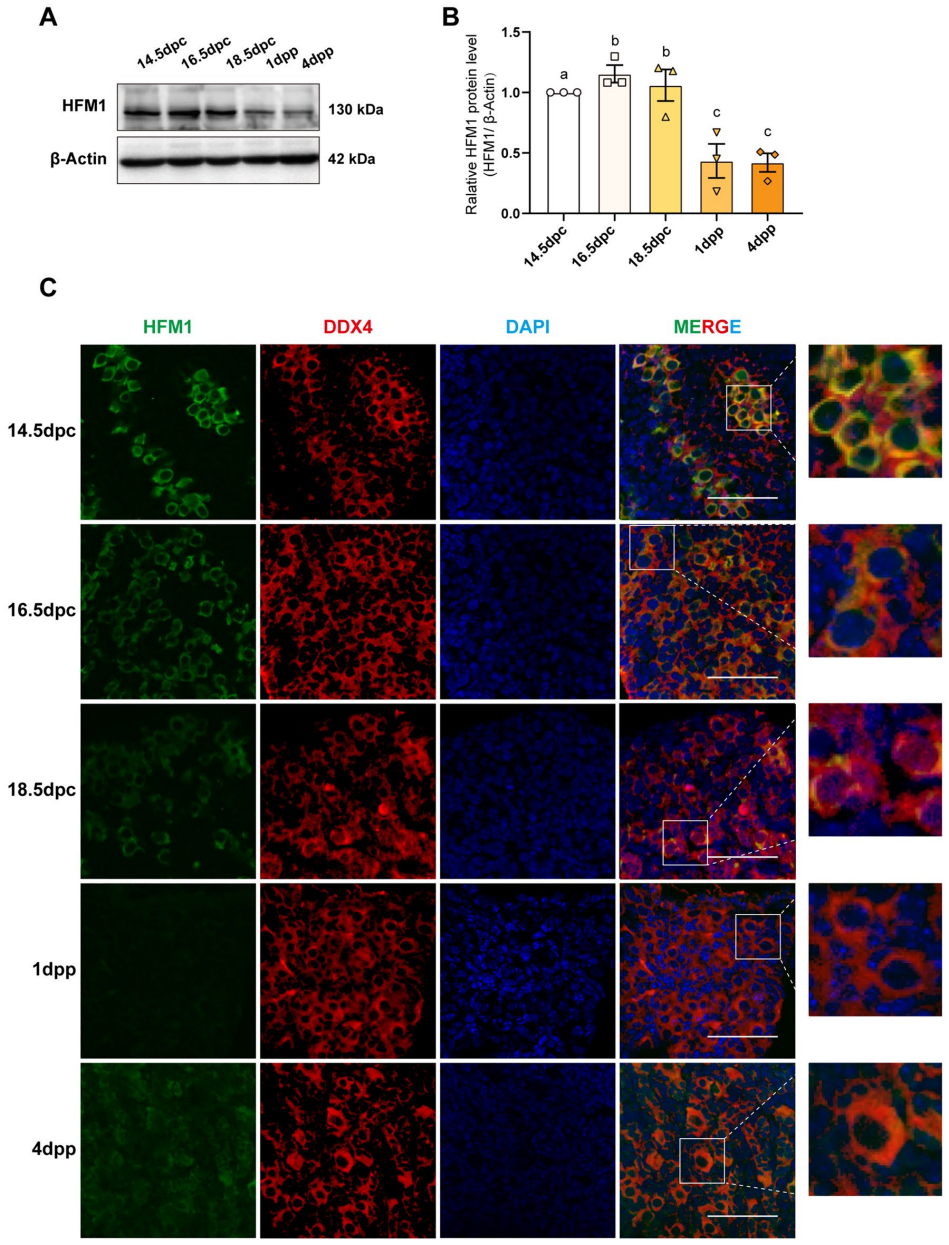
HFM1 predominantly expressed in the prenatal embryonic mouse ovary.** A** Immunoblotting staining showed that HFM1 was relatively highly expressed in the ovaries of embryonic mice at 14.5 days post coitum (dpc), 16.5 dpc, and 18.5 dpc, while the expression was significantly lower at 1 day post-partum (dpp) and 4 dpp after birth. β-actin was used as a loading control. **B** Quantification of HFM1 gray value. n = 3 biologically independent experiments. Data represented as mean ± standard error of the mean and the different letters (a-c) indicate the difference between the groups was statistically significant (two-sided ANOVA test), P (a, b) = ns, P (a, c) < 0.05, P (b, c) < 0.01. **C** HFM1 was mainly expressed in the cytoplasm of oocytes in mouse ovaries. Embryonic and neonatal mouse ovaries were stained for HFM1 (green) and germ cell-specific marker DDX4 (red). The nucleus was dyed with DAPI (blue). Scale bars: 50 μm

Immunoblotting was used to examine the protein expression level of HFM1 in embryonic and neonatal mouse ovaries, exploring the relationship between HFM1 and the first meiosis of oocytes. The mouse ovaries of 14.5 days post coitum (dpc), 16.5 dpc, and 18.5 dpc prenatal, and 1 day post-partum (dpp) and 4 dpp were collected. As shown in Fig. 1A, B, the protein expression level of HFM1 was significantly higher in the prenatal than that in the postnatal mouse ovaries.

To further investigate the function of HFM1, immunofluorescence was used to detect the localization of HFM1 in mouse ovaries. The co-staining of HFM1 (green) with the germ cell-specific marker DEAD-Box Helicase 4 (DDX4) (red) revealed that HFM1 was consistently highly expressed in the germ cells of mouse ovaries from 14.5 dpc to 18.5 dpc and primarily localized in the cytoplasm (Fig. 1C). During the postnatal period, the fluorescence intensity of HFM1 gradually weakened as the primordial follicle pool was established. The results showed that HFM1 had a comparatively high expression level in the prenatal period, suggesting that it may play a crucial role in the first meiotic prophase of oocytes as well as primordial follicle formation.

### Deficiency of *Hfm1* expression impeded oocyte meiotic process and cell survival in mouse ovaries

An adenovirus embedding *Hfm1*-RNA interference (AD-*Hfm1*i) was used to culture embryonic mouse ovaries of 14.5 dpc in vitro so as to understand the role of HFM1 in oocyte meiosis and primordial follicle formation. The ovaries transfected with adenovirus showed a strong green fluorescence after 4 days of in vitro culture, indicating successful transfection (Fig. S1A). Subsequent immunoblotting also confirmed that AD-*Hfm1*i significantly reduced the expression of HFM1 protein levels by half (Fig. S1B and S1C), implying AD-*Hfm1*i ovarian model was successfully constructed.

After successfully constructing the AD-*Hfm1*i ovarian model, the effect of HFM1 on germ cell numbers and oocyte meiotic prophase I were first investigated in mouse ovaries. Ovaries of 14.5 dpc were transfected with AD-*Hfm1*i or scrambled control and cultured for 4 days. The immunofluorescence and germ cell counts revealed the number of oocytes dramatically decreased after the treatment with AD-*Hfm1*i (Fig. S2A). The number of DDX4-positive oocytes in cultured ovaries was 8585 ± 1955 per ovary in the AD-*Hfm1*i group and 13,500 ± 1705 per ovary in the control group (*P* < 0.01; Fig. S2B), respectively.

Moreover, an increased TUNEL signal was observed in the 14.5 dpc AD-*Hfm1*i ovaries after 4-day cultivation with AD-*Hfm1*i (Fig. S2C) and the apoptotic rate was significantly higher in the AD-*Hfm1*i group (30.67%) than in the control group (7.25%) in ovarian sections (*P* < 0.05; Fig. S2D), suggesting a significant increase in the number of apoptotic cells. In addition, immunoblotting showed that the protein levels of cleaved-CASPASE3, P53, and P63 upregulated in HFM1-deficient embryonic ovaries (Fig. S2E), indicating increased apoptosis in the deficiency of HFM1. These results implied that the vital role of HFM1 in the survival of embryonic oocytes during meiotic prophase I and the deletion of HFM1 leads to extensive oocyte apoptosis.

Immunofluorescence was performed against Y box protein 2 (MSY2), which specifically presented in oocytes of the diplotene and afterward stages, to further evaluate the impact of HFM1 on the meiotic process of oocytes. The results showed that almost all the oocytes in the control group were positive for MSY2 on the fourth day of culture, while the number of MSY2-positive oocytes distinctly declined in the AD-*Hfm1*i group to about two thirds of that in the control group (Fig. S2F and S2G).

**Fig. 2:**
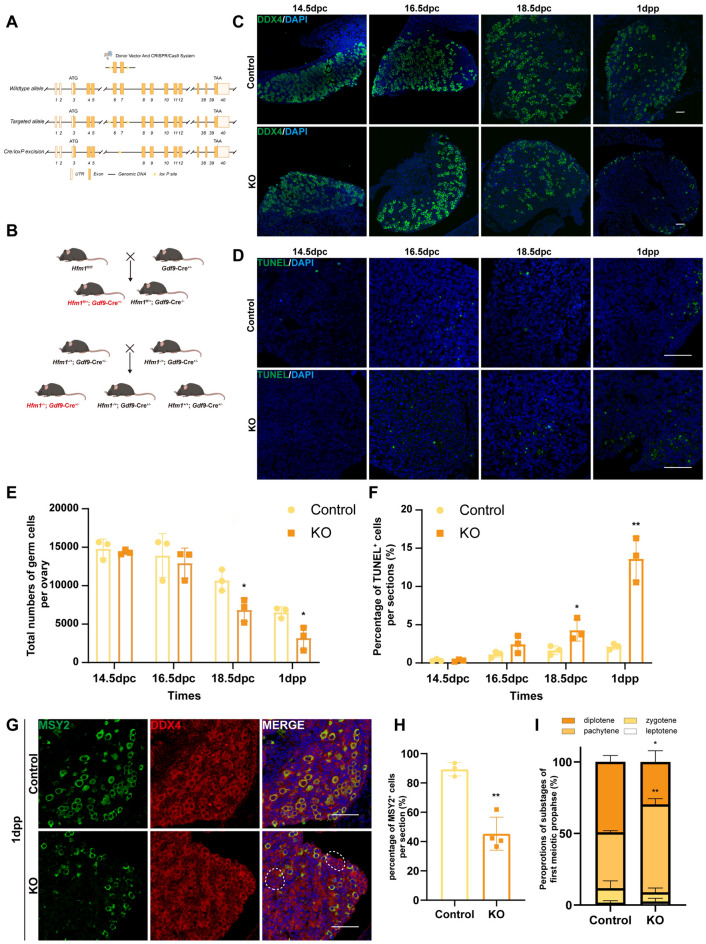
Deficiency of *Hfm1* expression impeded oocyte meiotic process and cell survival in mouse ovaries. **A** Engineered a conditional floxed allele for *Hfm1* and a *Cre*-mediated recombination to delete exons 6 and 7 of *Hfm1* in mice. **B** Schematic of the specific mating and breeding method to obtain systemic *Hfm1*-KO mice. **C** Deletion of *Hfm1* disrupted oocyte survival and early folliculogenesis in mice. Immunofluorescence staining showed ovaries of the Control and KO mice at the indicated developmental stages (14.5, 16.5, and 18.5 dpc and 1 dpp). Oocytes were stained with DDX4 (green). The nucleus was stained using DAPI (blue). Scale bars: 50 μm. **D** Apoptotic cells increased in KO ovaries compared with the Control ovaries with the development of oocytes. TUNEL signals (green) marked apoptotic cells, while the nucleus was stained using DAPI (blue). Scale bars: 50 μm. **E**, **F** Statistical analysis of total numbers of germ cells per ovary. (**E)** and the percentages of TUNEL^+^ cells per section (**F**) between Control and KO mice in the indicated developmental stages. ^*^P < 0.05, ^**^P < 0.01 (*t* test), *n* = 3. **G** First meiotic prophase in the *Hfm1-KO* mice at 1 dpp was arrested before the diplotene phase. The sections were stained with MSY2 (green) which specifically presented in oocytes of the diplotene and afterward stages and DDX4 (red). The nucleus was stained using DAPI (blue). The oocytes circled in the dashed line highlighted oocytes with no expression of MSY2. Scale bars: 50 μm. **H** Statistical analysis showed that the percentage of MSY2^+^ oocytes (number of cells both MSY2^+^ and DDX4^+^/number of cells DDX4^+^) per section decreased significantly following HFM1 deprivation. ^**^P < 0.01 (*t* test), *n* = 3. **I** Chromatin spread of 18.5 dpc ovaries showed that the KO group had more oocytes in the pachytene stage and fewer in the diplotene stage than the Control group. ^*^P < 0.05, ^**^P < 0.01 (*t* test), *n* = 3

The oocyte-specific deletion of the *Hfm1* mouse model was successfully established in a previous study [16]. To explore the function of HFM1 in oocytes before primordial follicle formation, a systemic knockout mouse model of HFM1 was established by mating and breeding the oocyte-specific deletion of the *Hfm1* mouse model (Fig. 2A, B). The *Hfm1*^*−/−*^*, GDF9-Cre*^+/ −^ female mice were regarded as *Hfm1* KO mice, while *Hfm1*^*−/*+^*, GDF9-Cre*^+/ −^ and *Hfm1*^+*/*+^*, GDF9-Cre*^+/ −^ females were regarded as control mice. Immunoblotting validated that hardly any HFM1 was expressed in the KO mice (Fig. S3A and S3B).

Proliferating cell nuclear antigen (PCNA) was assayed by immunofluorescence with the ovaries of 14.5 dpc, 16.5 dpc, 18.5 dpc, and 1 dpp in the control and KO mice (Fig. S4A). A considerable number of PCNA-positive germ cells were present in 14.5-dpc ovaries. As meiosis proceeded, the number of PCNA-positive germ cells gradually decreased until 1 dpp when nearly all germ cells showed no positive PCNA (Fig. S4B). Moreover, the apoptosis and survival of germ cells were also studied in *Hfm1* KO mice. Similarly, immunofluorescence was performed on the ovaries of 14.5 dpc, 16.5 dpc, 18.5 dpc, and 1 dpp mice. The results revealed that as the meiotic process of oocytes advanced, the number of apoptotic cells in the KO mice increased significantly and the number of oocytes decreased significantly compared with those in the control mice (Fig. 2C, D). The number of oocytes in the KO mice decreased by half at 1 dpp compared with that in the control group (Fig. 2E). Similarly, the rate of cell apoptosis increased significantly in the KO mice (Fig. 2F).

Dual-color immunofluorescence of 1 dpp ovaries in KO and control mice with DDX4 and MSY2 showed results comparable to those in AD-*Hfm1*i ovaries (Fig. 2G). Most of the 1-dpp oocytes in the control demonstrated MSY2 positivity, while only 50% of oocytes showed MSY2 positivity in the Hfm1-KO group, exhibiting a statistically significant difference (Fig. 2H). We then performed chromatin spread and immunofluorescence of the axial element, synaptonemal complex protein 3 (SYCP3), to analyze the meiotic process in 18.5-dpc ovaries (Fig. S3C). Half of the oocytes in the control mice were in the diplotene stage (49.17%), whereas only one third of oocytes in the KO mice progressed to the diplotene stage Fig. 2I.

### Depletion of *Hfm1* expression damaged the repair of DNA double-strand breaks and synaptonemal complex formation in *Hfm1*-KO mice

**Fig. 3:**
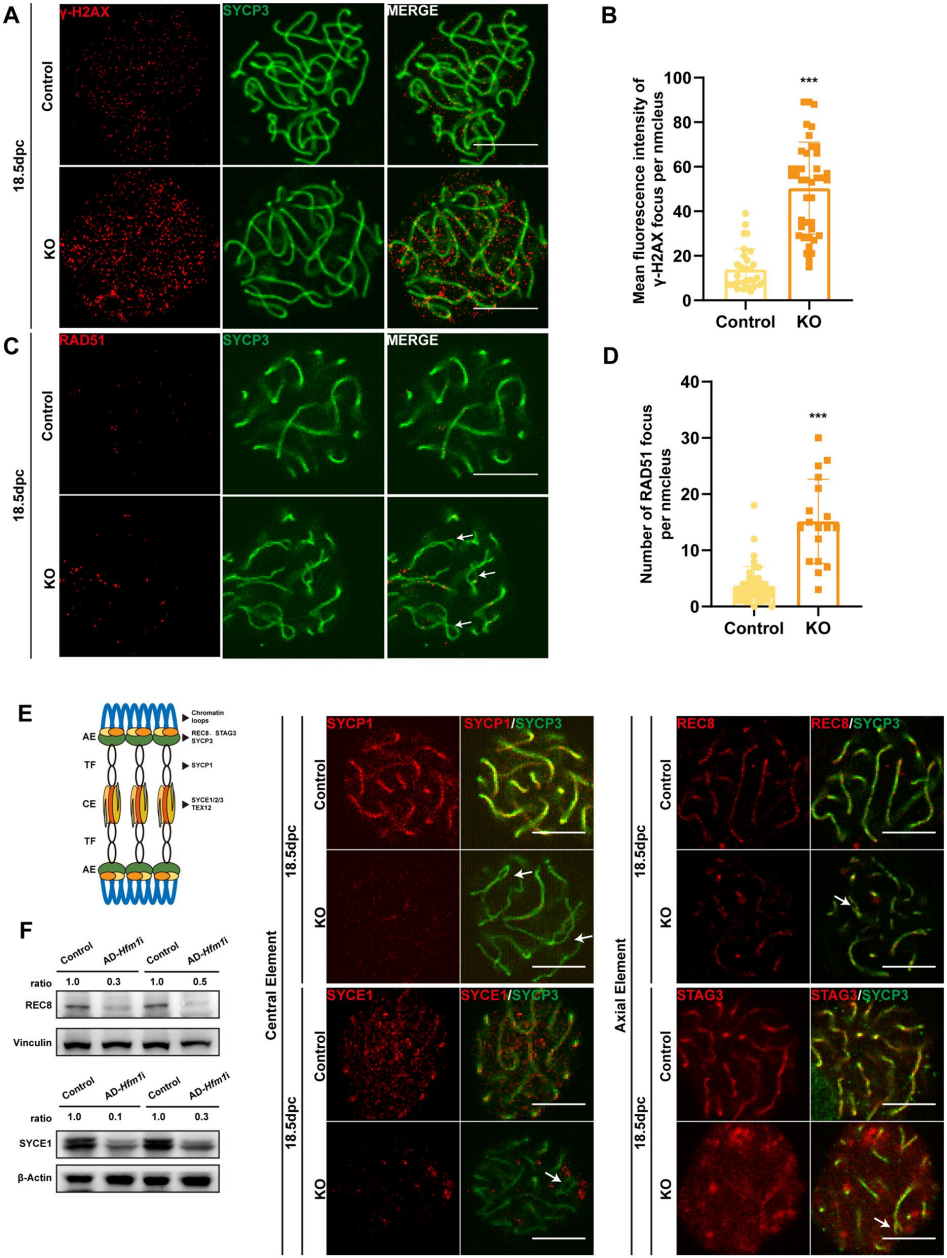
Depletion of *Hfm1* expression damaged the repair of DNA double-strand breaks and synaptonemal complex formation. **A**, **B** Deletion of *Hfm1* caused DSB repair deficiency in embryonic mouse ovaries. (**A)** Immunoblotting staining of the meiotic spread showed repaired or unrepaired DSBs in 18.5-dpc Control or KO ovaries. γ-H2AX (red) indicates unrepaired DSB sites. SYCP3 (green) demonstrates axial elements. Scale bars: 10 μm. (**B)** Statistical analysis showed that the mean fluorescence intensity of γ-H2AX on chromosomes per nucleus increased significantly following HFM1 deprivation. ^***^P < 0.001 (*t* test), WT: *n* = 31; KO: *n* = 41. **C**, **D** Deletion of *Hfm1* resulted in the ectopic expression of RAD51. (**C**) Immunoblotting staining showed normal or ectopic RAD51 (DNA break repair protein) focus in 18.5-dpc Control or KO ovaries. Oocyte chromosomes were co-stained with RAD51 (red) and SYCP3 (green). Scale bars: 10 μm. (**D**) Statistical analysis showed that the number of RAD51 foci on chromosomes per slide increased significantly following HFM1 deprivation. ^***^P < 0.001, WT: *n* = 42; KO: *n* = 19. **E** Schematic demonstrating the synaptonemal complex during the first meiotic prophase (left). Deletion of *Hfm1* impacted the formation of the synaptonemal complex. Immunoblotting staining of the meiotic spread showed abnormal expression of synaptonemal complex proteins SYCP1, SYCE1, REC8, and STAG3. Arrows demonstrated loose bivalent chromosome (right). Scale bars: 10 μm. **F** Immunoblotting showed significantly reduced expression in REC8 and SYCE1 of KO group (n = 3). β-Actin or Vinculin was used as a loading control

Evidence supports that the homologous recombination of chromosomes in the meiotic prophase I originates from DNA double-strand breaks (DSBs) [21]. The immunofluorescence against DNA damage repair marker protein γ-H2AX and DNA break repair protein RAD51 was performed on the spread chromosome of 18.5-dpc mouse ovaries. The deletion of Hfm1 sustained γ-H2AX focus on the oocyte chromosomes, indicating the presence of unrepaired DSBs on the chromosomes (Fig. 3A). More chromosomes with abnormal γ-H2AX signals were observed in the KO mice compared with the control mice (Fig. 3B). Meanwhile, the RAD51 focus on chromosomes was significantly more in the HFM1-deficient oocytes (14.6 ± 7.6) than in the control oocytes (3.7 ± 3.4, Fig. 3C, D). Also, the fluorescence of SYCP3, a component of the synaptonemal complex (SC), in the KO mice seemed to diminish compared with that in the control mice. To figure out whether synaptonemal complexes is affected, key component proteins (SYCP1, SYCE1, REC8, and STAG3) of the synaptonemal complex were also examined by immunofluorescence. The fluorescence intensity of key proteins, such as SYCP1, REC8, SYCE1, and STAG3, was significantly reduced in HFM1-deficient oocytes (Fig. 3E). Immunoblotting also showed significantly reduced expression in REC8 and SYCE1 of knockdown group, which proponent of above result (Fig. 3F). The results suggested that synaptic defects were evident in the chromosomes of the KO mice and HFM1 deletion led to the disruption of synaptonemal complex formation and thus disorders of DNA break repair and chromosome synapsis.

### HFM1 may regulate the first meiotic prophase of the oocyte by interacting with FUS

**Fig. 4:**
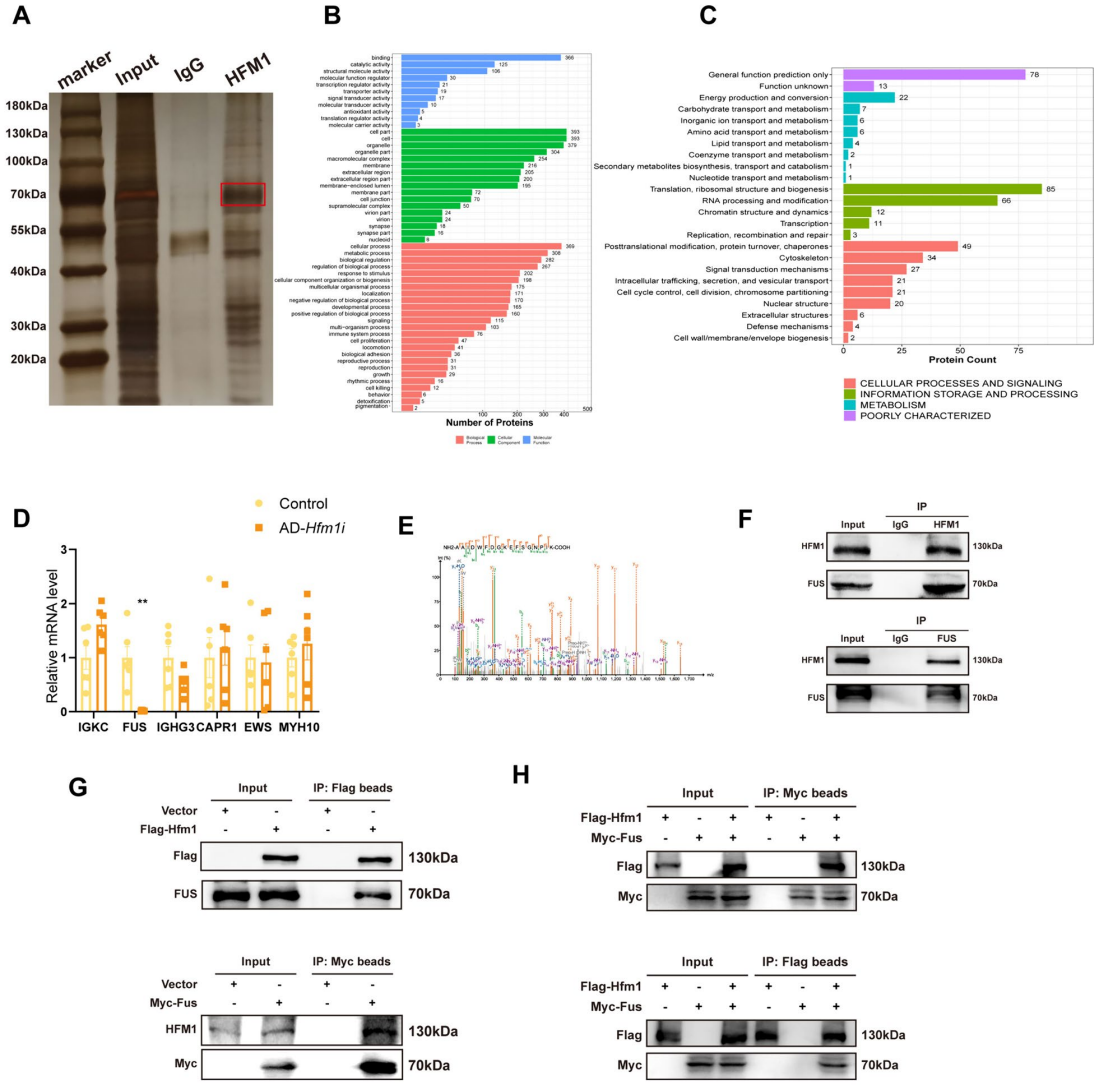
HFM1 and FUS interacted with each other.** A** Co-IP and silver staining showed the proteins bound to HFM1. **B**, **C** Functional analysis of enriched genes by Co-IP. Gene Ontology (GO) analysis (**B**) and eukaryotic orthologous groups (KOGs) analysis (C) of enriched genes described the Molecular Function, Cellular Component and Biological Process of the enriched genes. **D** Real-time PCR showed that the interference of HFM1 expression decreased the expression of FUS, but not IGKC, IGHG3, CAPR1, EWS, and MYH10. ^**^P < 0.01, *n* = 6. **E** Mass spectrometry (MS) analysis to determine which proteins bound to HFM1 showed FUS to be an interacting protein. **F** Endogenous protein interactions of HFM1 and FUS were assessed in embryonic mouse ovary lysates by immunoprecipitation with anti-HFM1 or anti-FUS and evaluated using immunoblotting with indicated antibodies. IgG was used as a negative control. **G**, **H** Exogenous protein interactions demonstrated in HEK 293 T cells. HEK 293 T cells transfected with indicated plasmid (Flag-tagged HFM1 plasmid, Myc-tagged FUS plasmid and plasmid vector, separately) and treated with proteasome inhibitor MG132 (10 μM). Cells were lysed with NP-40 and analyzed using Co-IP with Flag or Myc beads followed by immunoblotting

The Co-IP following silver stain was performed on mouse ovaries at 17.5 dpc to identify the targets of HFM1, revealing that HFM1-IP-enriched proteins were concentrated at around 70 kDa (Fig. 4A). Totally, 445 proteins were identified by mass spectrometry (MS) (Table. S1). GO analysis suggested genes mainly enriched in “bind”, “cell part”, and “cellular process” (Fig. 4B). KOG analysis showed genes enriched in “translation, ribosomal structure and biogenesis”, “translational modification, protein turnover, chaperones” (Fig. 4C). We measured the mRNA level of the most abundant proteins, IGKC, FUS, IGHG3, CAPR1, EWS, and MYH10, among all the putative proteins. FUS was significant decreased after knockdown of Hfm1 in ovaries (Fig. 4D). FUS, identified as a putative HFM1-interacting protein by MS, has been reported to regulate DSB repair [22, 23] (Fig. 4E). Further Co-IP combined with immunoblotting was performed in embryonic mouse ovaries for validation the co-precipitation of endogenous HFM1 with FUS and vice versa (Fig. 4F). In addition, an in vitro binding assay demonstrated that exogenously expressed HFM1 interacted with FUS (Fig. 4G, H). Together, these data demonstrated that HFM1 could interact with FUS.

### HFM1 inhibited the ubiquitination degradation of FUS and the cytoplasmic–cytosolic localization of FUS

**Fig. 5:**
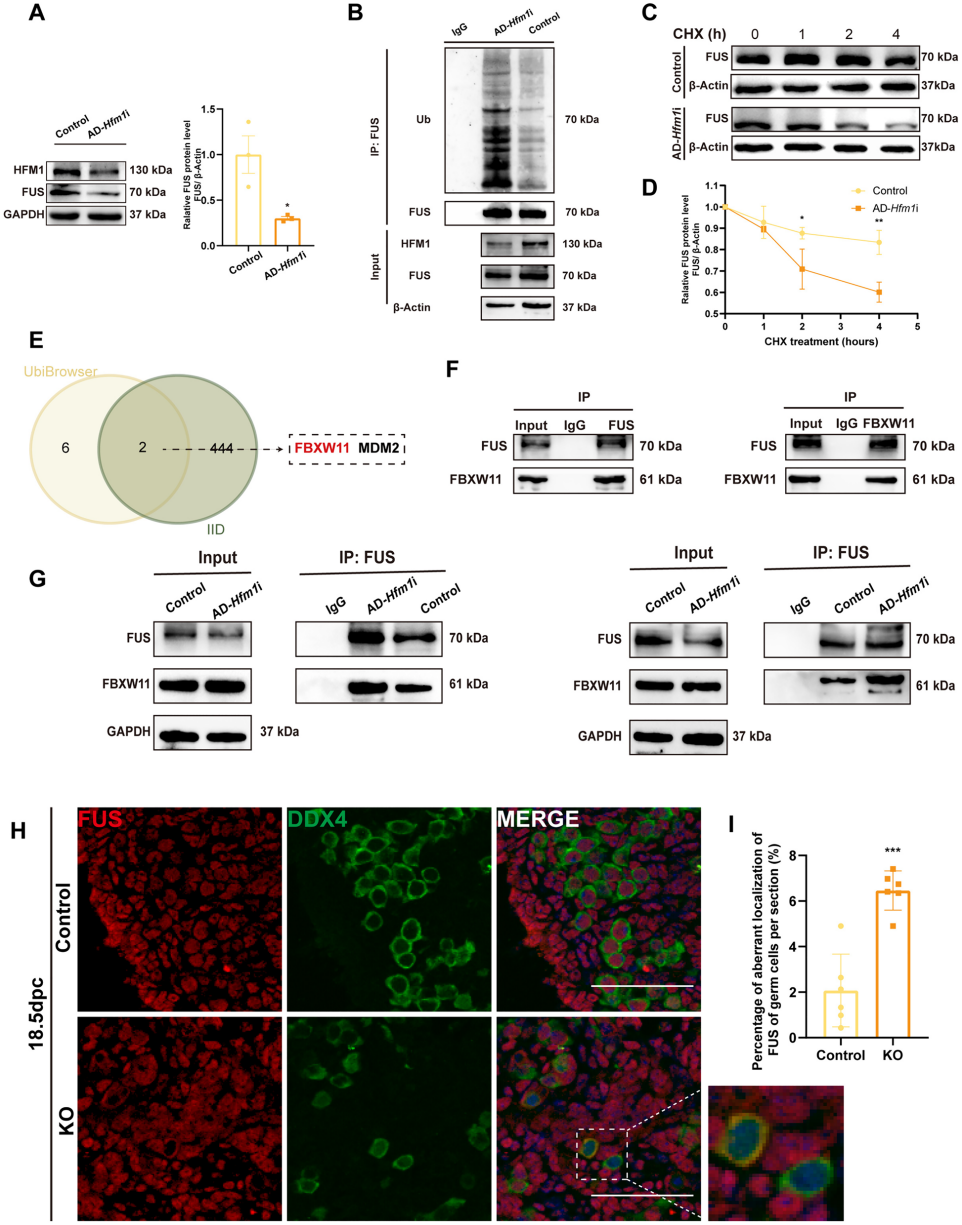
HFM1 acted on the ubiquitination and degradation of FUS and the cytoplasmic–cytosolic localization of FUS.** A** HFM1 silencing led to a decrease in FUS protein expression (^*^P < 0.05, n = 3). GAPDH was used as a loading control. **B** Lysates from embryonic ovaries transfected with control or AD-*Hfm1*i, followed by treatment with MG132 before harvest, were immunoprecipitated and examined with indicated antibodies. Quantification of relative ubiquitin-FUS levels showed that the ubiquitination level of FUS increased after HFM1 knockdown. **C**, **D** Embryonic ovaries were cultured with control or AD-*Hfm1*i, treated with cycloheximide (CHX, 100 μg/mL), and collected for immunoblotting analysis at the indicated time points (C). Quantification of FUS band intensity was presented (D). ^*^P < 0.05, ^**^P < 0.01 (*t* test), *n* = 3. **E** Venn diagram showed that FBXW11 and MDM2 may be the potential ligating (E3) enzymes during the ubiquitination of FUS using UbiBrowser database (http://ubibrowser.bio-it.cn/ubibrowser/) and the Integrated Interactions Database (http://iid.ophid.utoronto.ca/search_by_proteins/). **F** Endogenous protein interactions of FBXW11 and FUS were assessed in embryonic mouse ovary lysates by immunoprecipitation with anti-FBXW11 or anti-FUS and evaluated using immunoblotting with indicated antibodies. IgG was used as a negative control. **G** Lysates from ovaries transfected with control or AD-*Hfm1i* were collected for Co-IP. The binding of FUS and FBXW11 increased with the knockdown of *Hfm1*. **H**, **I** HFM1 maintained the nuclear localization of FUS in embryonic mouse oocytes. 18.5-dpc embryonic mouse ovaries were stained for FUS (red) and germ cell-specific marker DDX4 (green), while the nucleus was stained using DAPI (blue). The area boxed by dotted line was the oocytes with aberrant localization of FUS (H). Scale bars: 50 μm. Statistical analysis showed the number of germ cells with aberrant-localized FUS per section (I). ^***^P < 0.001 (*t* test), *n* = 6

As shown in Fig. 5A, the deletion of HFM1 led to a reduction in the FUS protein level. Further detection of the ubiquitination levels in FUS by adding the protease inhibitor MG132 revealed that the knockdown of HFM1 efficiently elevated the ubiquitination level of FUS (Fig. 5B). Then, the impact of HFM1 depletion on the stability of the FUS protein in the cultured ovaries treated with cycloheximide (CHX), a protein synthesis inhibitor, was examined. The half-life of FUS protein was markedly reduced in the HFM1 low-expression group compared with the control group, and the degradation level of the FUS protein was much higher at a same time point (Fig. 5C, D).

The ubiquitination degradation of protein requires the activation of ubiquitin (Ub) by an activating (E1) enzyme and transfer onto a conjugating (E2) enzyme. The ligating (E3) enzyme then attaches to the Ub bound to E2 to the substrate protein, and subsequently the proteasome specifically binds to and degrades the Ub-loaded protein [24]. We intersected eight potential E3 ligases of FUS from the UbiBrowser Database (http://ubibrowser.bio-it.cn/ubibrowser/) and 446 putative FUS interaction proteins from the Integrated Interactions Database (http://iid.ophid.utoronto.ca/search_by_proteins/) to identify ligases in the ubiquitination of FUS. Two possible E3 ligases, FBXW11 and MDM2, were obtained (Fig. 5E). Co-IP using fetal ovaries verified the mutual binding between FUS and E3 ligases, FBXW11 and MDM2 (Fig. 5F and Fig. S5A). Subsequently increased binding of FUS with FBXW11 was observed using Co-IP in the absence of HFM1, indicating an increased FBXW11-mediated ubiquitination degradation level of FUS (Fig. 5G) while binding of FUS and MDM2 did not changed significantly with the depletion of HFM1 (Fig. S5B).

In addition, the immunofluorescence showed that the localization of FUS was altered in part of embryonic mouse ovaries after knockout of *Hfm1* at 18.5 dpc. FUS was widely expressed in the nuclei of oocytes in the control embryonic ovaries, but was aberrantly located in the cytoplasm of some oocytes in the ovaries of the KO mice (Fig. 5H, I). Interestingly, immunofluorescence of chromatin spread showed that HFM1 expressed not only in cytoplasm, but also in spots on chromosome axes (Fig. S5C). Consistent with that, HFM1 foci was found on chromosome axes in spermatocyte [25]. These results suggested that HFM1 contributed to maintaining the localization of FUS in the nucleus of oocytes during the meiotic prophase I.

### BRCA1 might be the target of the HFM1–FUS axis

**Fig. 6:**
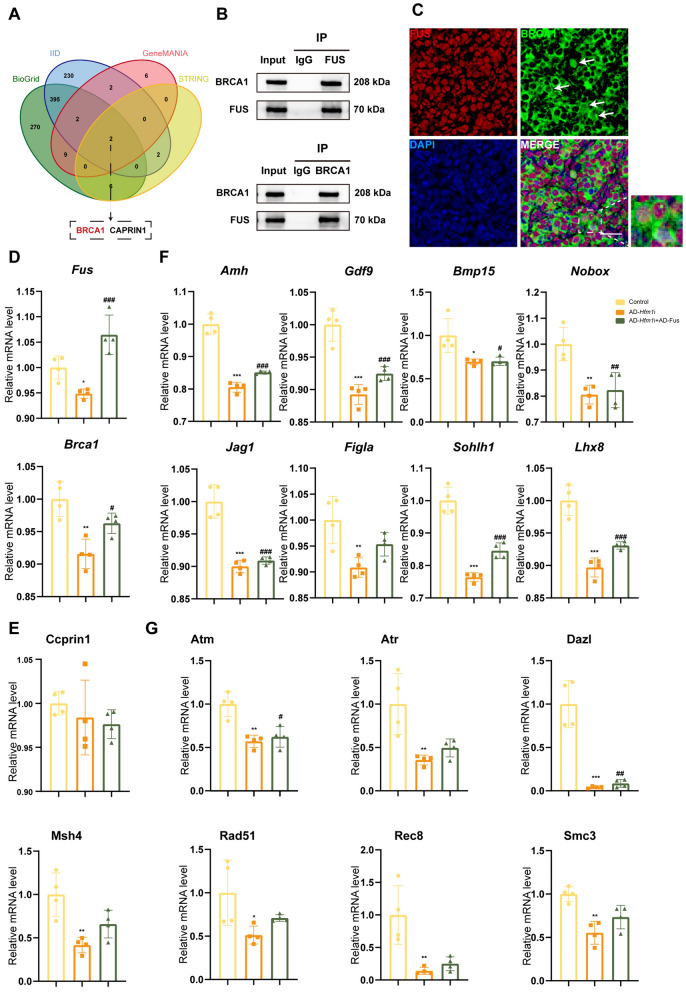
BRCA1 might be a possible target of the HFM1-FUS axis.** A** Venn diagram showed that BRCA1 and CAPRIN1 were the binding proteins of FUS using BioGrid, IID, GeneMANIA, and STRING. **B** Lysates from embryonic ovaries were immunoprecipitated and examined with indicated antibodies to assess the endogenous protein interactions of BRCA1 and FUS. **C** BRCA1 co-localized with FUS in some oocytes of 18.5-dpc embryonic mouse ovaries. BRCA1 was stained with green, and FUS was stained with red. The nucleus was stained using DAPI (blue). The oocytes in which BRCA1 co-localized with FUS are highlighted in dashed boxes or pointed by arrows. Scale bars: 10 μm. **D** Real-time PCR showed that the interference of HFM1 expression decreased the expression of *Brca1*, and the overexpression of FUS restored the mRNA level of *Brca1*. **E** Real-time PCR showed interference of HFM1 expression would not change the expression of ccprin1. **F**, **G** HFM1 regulated the expression of oocyte development-related factors (F) and meiosis-related factors (G) by affecting FUS-BRCA1. Real-time PCR showed that the interference of HFM1 expression decreased the expression of those genes, while the overexpression of FUS restored the expression of these genes appropriately. Compared with the control group, ^*^P < 0.05, ^**^P < 0.01, ^***^P < 0.001; compared with the AD-Hfm1-RNAi group, ^#^*P* < 0.05, ^##^*P* < 0.01,.^###^*P* < 0.001, *n* = 4

Two putative FUS-interacting proteins, cell cycle–associated protein 1 (CCPRIN1) and breast cancer susceptibility gene 1 (BRCA1), were obtained by the intersection of the FUS-interacting proteins retrieved from Genemania Database (http://genemania.org/), BioGrid Database (https://thebiogrid.org/), STING Database (https://string-db.org), and Integrated Interactions Database (http://iid.ophid.utoronto.ca/search_by_proteins/) (Fig. 6A). The FUS-interacting proteins are listed in Supplementary Table 5. Further, AD-*Hfm1i* and adenovirus *Fus* (AD-*Fus*) were used to transfect ovaries at 14.5 dpc. After 4 days of culture, the mRNA levels of key genes for meiosis and oocyte development were measured using real-time PCR. As shown, the mRNA level of *Brca1* reduced, accompanied by the absence of HFM1, but increased after the overexpression of FUS, while *ccprin1* showed little change (Fig. 6D, E). The important role of BRCA1 in DSB repair and recombination has been reported, and its dysfunction is associated with POI [26, 27]. Co-IP in embryonic ovaries verified the combination of FUS with BRCA1 (Fig. 6B). Additionally, the immunofluorescence showed that BRCA1 also co-localized with FUS in the nucleus of some oocytes (Fig. 6C). Furthermore, the mRNA levels of oocyte development–related genes *Gdf9*, *Bmp15*, *Jag1*, *Figla*, *Nobox*, *Sohlh1*, *Amh*, and *Lhx8* (Fig. 6F) and meiosis-related genes *Atm*, *Atr*, *Dazl*, *Msh4*, *Rad51*, *Rec8*, and *Smc3* (Fig. 6G) significantly decreased after the depletion of HFM1, but they were partially restored after the overexpression of FUS.

## Discussion

**Fig. 7:**
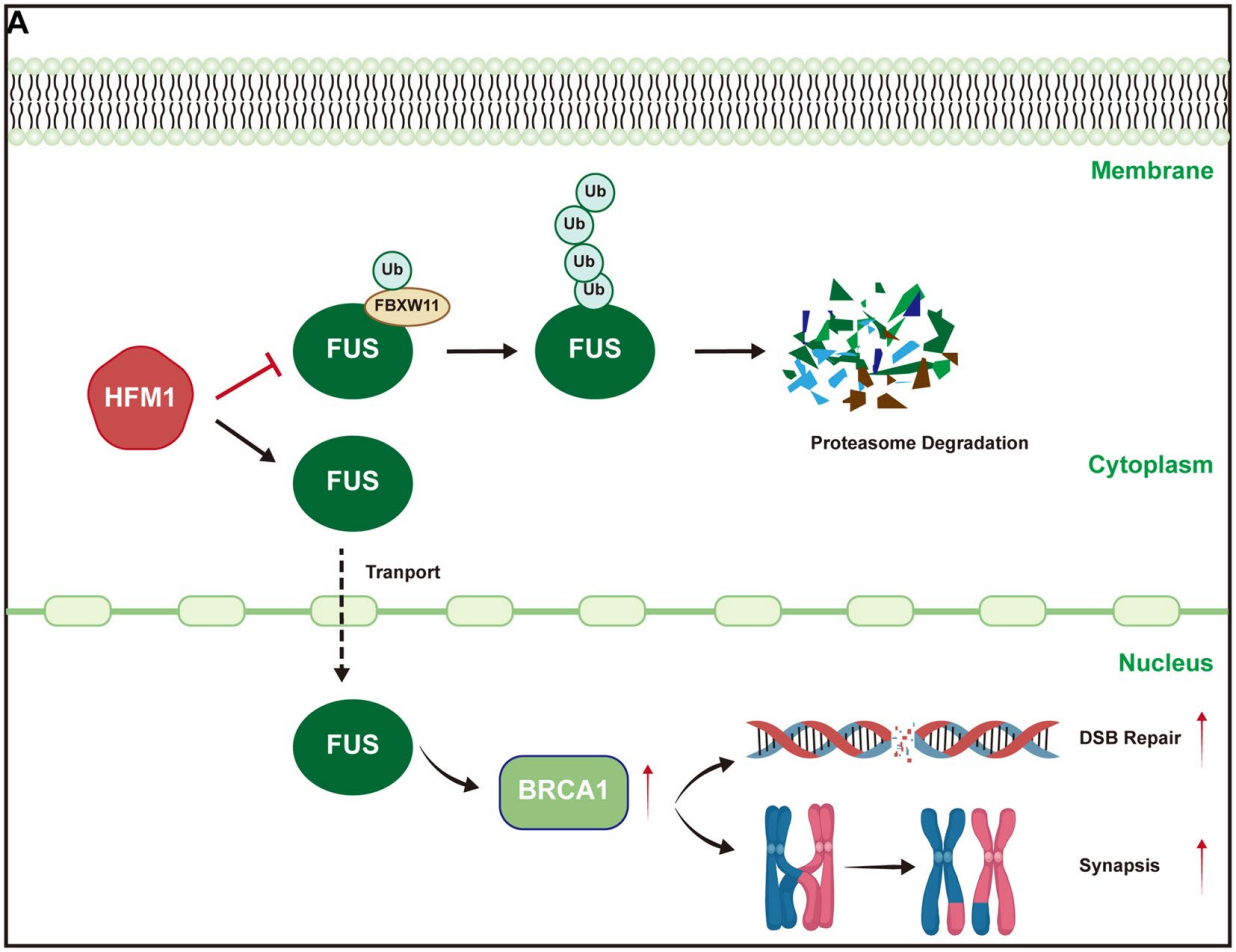
Schematic model of HFM1 functioning in oocytes during the first meiotic prophase. HFM1 regulated E3 ubiquitin ligase FBXW11-mediated ubiquitination degradation of FUS and maintained the nuclear localization of FUS in oocytes, thereby regulating the expression of BRCA1 and affecting oocyte DSB repair and synapsis

The failure to establish a perinatal primordial follicle pool leads to ovarian insufficiency or even infertility [28, 29]. In the present study, an in vitro ovary culture system and a knockout mouse model were used to explore the role of HFM1 in the meiotic prophase I of oocytes and the establishment of the perinatal primordial follicle pool. It was found that HFM1 deprivation led to cell apoptosis in ovaries and a significant reduction in the number of oocytes, resulting in a diminution of the primordial follicle pool. Further studies revealed that HFM1 was engaged in modulating E3 ubiquitin ligase FBXW11-mediated ubiquitination degradation of FUS protein and maintaining its intranuclear localization. This affected BRCA1 expression, which participated in synaptonemal complex formation and DSB repair during the normal developmental process of the meiotic prophase I of oocytes (Fig. 7). Our study provided insights into the function of HFM1 in oocyte meiotic prophase I and primordial follicle formation, thus providing evidence for the risk identification and early intervention of POI in clinical practice.

The role of HFM1 in meiotic prophase I has been reported in *Saccharomyces cerevisiae*, *Arabidopsis*, and other organisms, and the mutations in Mer3, a homolog of HFM1, could cause delayed repair of DSBs, defective formation of cross-over, abnormality of chromosome synapsis, and mis-segregation of chromosomes [30–33]. The cross-species failure of the assembly of the synaptonemal complex suggested that the requirement on Mer3 was highly conserved in evolution and was important in meiosis. *Hfm1*-KO mice spermatocytes exhibited defects in cross-over formation and homologous recombination in male [15]. And recent studies showed the mutations in the *HFM1* gene were associated with the occurrence of POI in women [34, 35] and oligospermia/ azoospermia in men [36, 37]. In those cases, the spermatocytes of patients are arrested in the pachytene stage, which is consistent with our results.

Additionally, the meiotic process of the *Hfm1*-KO mice was arrested before the diplotene stage in this study. Previous studies showed that the germ cells which unable to complete homologous recombination and repairment of disruption in the synapsis would result in an increase in germ cell apoptosis and a reduction in cell numbers [38, 39]. Our results were consistent with these findings. In this study, the formation of the synaptonemal complex was impaired, and DSB repair remained incomplete in the *Hfm1*-KO mice. The expression of synaptonemal complex proteins SYCP1, SYCE1, REC8, and STAG3 was obviously aberrant, and the γ-H2AX and RAD51 focus persisted on chromosomes. In particular, the segregation of SYCP3 as a scaffold in some homologous chromosomes was detected, indicating the essential role of HFM1 in the formation of the synaptonemal complex and the repair of DSBs. Therefore, we believed that HFM1 deletion could lead to a defect in the first meiosis in mice, thus causing an increase in oocyte apoptosis and the inability to establish a normal primordial follicle pool, leading to POI or even infertility.

To explore the targets of HFM1 in the meiotic prophase I of oocytes, HFM1-IP with MS was conducted. 445 proteins were identified by MS in total. Among the top five most abundant proteins, IGKC, GCAB, FUS, ACTB, IGHG3, FUS has been reported to work as important effectors of the cellular DNA damage response [22, 23]. Fused in sarcoma/translocated in liposarcoma (FUS/TLS) is a DNA/RNA-binding protein regulating transcription, RNA splicing and transport [40]. A FUS-dependent liquid–liquid-phase separation has been reported to play a key role in activating the DNA damage response and the correct assembly of the DSB repair complex in HeLa Cells [23]. FUS could serve as a target for ATM as well as PARP downstream to respond to DNA damage [41, 42]. Like our findings in *Hfm1-KO* mice, the mutations in *Fus* led to infertility in heterozygous male mice with increased numbers of mismatched chromosomes and abnormal synapsis in spermatocytes [43]. We further showed that HFM1, acting as a repressor, regulated the ubiquitinated degradation of FUS mediated by FBXW11 to influence the stability of FUS. Furthermore, FUS is a nuclear protein with nucleoplasm shuttle capability, and defects in shuttling can lead to degenerative neurological diseases [44, 45]. We found, using the immunofluorescence of embryonic ovaries, that FUS was mislocalized in the cytoplasm of oocytes in the *Hfm1-KO* mice. Therefore, we thought that the mislocalization of FUS might result from the deletion of HFM1, which associated with the disorder of the first meiotic process of oocytes.

Interestingly, we found that BRCA1 might be the target of FUS BRCA1 was identified as a critical player in the maintenance of genomic stability [46]. BRCA1 was reported to co-localize with RAD51 at the DNA damage repair focus and interact with a range of molecules, including BARD1 (via its N-terminal ring finger domain), DNA repair enzymes (mainly via its central domain), and transcriptional activators (primarily via two tandem BRCA1 C-terminal, or BRCT, motifs), thereby repairing DSBs and promoting the homologous recombination of chromosomes [47–49]. It is reported that women carrying BRCA1 mutations tended to develop infertility or POI due to the accumulation of DNA damage which negatively impacting ovarian reserve [50]. A reduction of the ovarian reserve was also speculated in some BRCA1/2 mutant patients who got an earlier mean age of menopause, or a reduced presence of antral follicles, or a decrease in serum AMH level [26]. Thus, the detailed mechanism of BRCA1 and FUS in the establishment of primordial follicular pool is worth studying.

## Conclusions

Collectively, HFM1 regulates meiotic progression in the embryonic ovary by regulating the E3 ubiquitin ligase FBXW11-mediated ubiquitination degradation of the FUS protein and maintaining its intranuclear localization. It subsequently affects BRCA1 expression, which participates in synaptonemal complex formation and DSB repair. Our study identified HFM1 as one of the key proteins for the first meiotic prophase process and oocyte survival in mouse oocytes. Although, more studies are warranted to elucidate the role of HFM1 in the pathogenesis of POI in clinical practice, we hope our findings provided better understanding of the etiology of POI and other reproductive disorders caused by the obstruction of primordial follicle formation, and ultimately helped establish prevention and treatment strategies for POI.

### Supplementary Information

Additional file 1: Supplementary Tables 1. Proteins identified by mass spectrometry (MS). Supplementary Tables 2. List of primary antibodies used for immune detection. Supplementary Tables 3. List of primers use for knockdown adenovirus HFM1 interfere. Supplementary Tables 4. List of primers used for quantitative real-time PCR. Supplementary Tables 5. The FUS-interacting proteins from different databases.

Additional file 2: Figure S1. Ad-*Hfm1*i significantly reduced the protein level of HFM1 in the prenatal embryonic mouse ovary. (A) 14.5 dpc ovaries were cultured with adenovirus for 4 days. Immunofluorescence staining showed adenovirus could successfully transfect into ovaries. DIC indicated differential interference contrast, and GFP indicated cells in ovaries were infected with adenovirus. Scale bars: 10μm. (B and C) Immunoblotting analysis of HFM1 protein level following HFM1 inhibition. β-Actin was used as a loading control. Quantification of HFM1 gray value. *p<0.05 (t-test), n=3. Figure S2. Knockdown of *Hfm1* expression inhibited meiotic process and cell survival in mouse ovaries. (A and B) Inhibition of *Hfm1* by AD-*Hfm1*i led to dramatic oocyte loss in fetal ovaries. 14.5 dpc embryonic mice ovaries were cultured with AD-*Hfm1*i *in vitro* for 4 days and the oocytes were counted. Oocytes were stained with DDX4 (green) and the nucleus was stained with DAPI (blue). Statistical analysis showed that the total number of oocytes decreased following the decrease of HFM1 expression compared to the control group. **p<0.01 (t-test), n=6. Scale bars: 50μm. (C and D) Inhibition of *Hfm1* caused severe cellular apoptosis in the fetal ovaries. TUNEL signals (green) corresponded to apoptotic cells. The nucleus was stained by DAPI (blue). Statistical analysis showed a significant increase in apoptosis, *p<0.05 (t-test), n=3. Scale bars: 50μm. (E) Immunoblotting analysis of Cleaved-CASPASE3, P63, and P53 proteins level following HFM1 reduction. β-Actin or GAPDH were used as loading control. (F and G) Oocytes at the diplotene stage were reduced following HFM1 inhibition. MSY2(green) marked oocytes entering the diplotene stage and DDX4 (red) marked germ cells. The nucleus was immunostained by DAPI (blue). The encircled areas by dotted line were MSY2^-^/DDX4^+^ germ cells. Statistical analysis showed that the percentage of MSY2^+^ oocytes (number of cells both MSY2^+^ and DDX4^+^/number of cells DDX4^+^) per section decreased significantly following HFM1 inhibition. **p<0.01 (t-test), n=4. Scale bars: 50μm. Figure S3. Hfm1-KO mouse model was successfully obtained. (A and B) Immunoblotting demonstrated little to no expression of HFM1 protein in the KO mice model. β-Actin was used as a loading control. Quantification of HFM1 gray value. **p<0.01 (t-test), n=3. (C) Representative examples of the phases of meiosis prophase I in mouse oocytes. chromosome spreads were immunolabeled for SYCP3 (green) and nucleus stained with the nuclear marker DAPI (blue). The prophase stages were defined as: leptotene, SYCP3 was distributed in a punctate and discontinuous way; zygotene, shorter and thicker chromosomes and classical tripartite synaptonemal complex structure at homologous pairing site; pachytene, maximal shortening and thickening of the paired homologous chromosomes; diplotene, separation of homologous chromosomes. Scale bars: 10 μm. Figure S4. Knockout of *Hfm1* had no impact on the proliferation of germ cells in embryonic ovaries. (A) Proliferation of germ cells showed no difference in KO ovaries compared with the control ovaries as the oocyte developed. PCNA signals (green) marked proliferating cells while the nucleus was stained by DAPI (blue). Scale bars: 50μm. (B) Statistical analysis showed that the percentage of PCNA^+^ germ cells (number of cells both PCNA^+^ and DDX4^+^/number of cells DDX4^+^) per section showed no difference between the control and KO group. p>0.05 (t-test), n=3. Figure S5. HFM1 did not affect the interaction between FUS and MDM2. (A) Endogenous protein interactions of MDM2 and FUS were assessed in embryonic mouse ovary lysates by immunoprecipitation with anti-MDM2 or anti-FUS and evaluated using immunoblotting with indicated antibodies. IgG was used as a negative control. (B) Lysates from ovaries transfected with control or AD-*Hfm1*i were collected for Co-IP. The binding of FUS and MDM2 was measured with the knockdown of* Hfm1*. (C) HFM1 co-localized with SYCP3 on chromosome axes in a dotted way. SYCP3 was stained with green, and HFM1 was stained with red. The nucleus was stained using DAPI (blue). Scale bars: 10 μm.

## Data Availability

All data generated or analyzed during this study are included in this published article and its supplementary information files.
